# Study of Glucose-6-Phosphate Dehydrogenase (G6PD) Deficiency and Genotype Polymorphism of G6PD B and G6PD (A+/A-) in Patients Treated for Plasmodium vivax Malaria in a Tertiary Care Hospital in North East India

**DOI:** 10.7759/cureus.11463

**Published:** 2020-11-12

**Authors:** Purnima Rajkhowa, Chandan Nath, Anirban Dutta, Ishita Misurya, Nalini Sharma, Bhupen Barman, Chubalemia Longkumer, K G Lynrah, Devajit Sarmah, Alice Ruram

**Affiliations:** 1 Department of Microbiology, Silchar Medical College, Silchar, IND; 2 Department of Biochemistry, North Eastern Indira Gandhi Regional Institute of Health and Medical Sciences, Shillong, IND; 3 Department of Medicine, Dr. NMB Baruah Nursing Home, Nalbari, IND; 4 Department of Medicine, All India Institute of Medical Sciences, Jodhpur, IND; 5 Department of Obstetrics and Gynecology, North Eastern Indira Gandhi Regional Institute of Health and Medical Sciences, Shillong, IND; 6 Department of Medicine, North Eastern Indira Gandhi Regional Institute of Health and Medical Sciences, Shillong, IND; 7 Department of Biochemistry, Woodland Hospital, Shillong, IND; 8 Department of Biochemistry, Rajarshi Dashrath Autonomous State Medical College, Ayodhya, IND

**Keywords:** glucose-6-phosphate-dehydrogenase deficiency (g6pd), malaria, north east india, g6pd genotype, plasmodium vivax, g6pd b, g6pd a(+), g6pd a(-)

## Abstract

Introduction

Glucose-6-phosphate dehydrogenase (G6PD) enzyme deficiency is the most common enzymopathy in humans, and its distribution has been historically described to be closely associated with that of malaria. North East India provides optimal conditions for transmission of malaria and bears a considerable burden of *Plasmodium vivax* (*P. vivax*) malaria. Primaquine, a mainstay in the treatment of vivax malaria, may trigger episodes of acute hemolysis in patients with G6PD deficiency. The present study sought to delineate the frequency and genotypes of G6PD deficiency among patients suffering from vivax malaria infections.

Methods

Blood specimens from 80 individuals diagnosed with vivax malaria underwent enzyme assay for G6PD deficiency. Samples with deficient phenotype underwent isolation of DNA using a genomic DNA isolation kit (Qiagen India Pvt. Ltd., New Delhi, India). The genomic DNA underwent amplification, serial denaturation, annealing, extension, final extension followed by digestion with restriction endonucleases Nla III and Fok I. The digested products were subjected to horizontal agarose electrophoresis for the separation of digested fragments. Samples without nucleotide 376 adenine→guanine (A→G) mutation were classified as G6PD B. Those with the mutation were further classified into G6PD A(+) and G6PD A(-) based on the presence of Nla III site.

Results

Twenty-seven out of 80 individuals (33.75%) with *P. vivax* malaria were found to have G6PD deficiency, of which a majority (n=24) had G6PD B genotype. Three individuals had Asparagine→Aspartic Acid mutation at position 376 (A→G), of which G6PD A(+) and G6PD A(-) were present in two and one cases, respectively.

Conclusion

G6PD deficiency was noted in about a third of patients with vivax malaria. Since primaquine therapy is contraindicated in this group of patients, there is a rationale for looking into screening patients with vivax malaria from the region prior to primaquine therapy. Further large scale studies may substantiate this and help in better genotypic and geographic characterization of G6PD deficiency in the region.

## Introduction

Glucose-6-phosphate dehydrogenase (G6PD) deficiency is the most common human enzyme deficiency, affecting an estimated 400 million people globally [1]. G6PD is a key component in the pentose phosphate pathway, employed by erythrocytes to handle oxidative damage [2]. After invading host erythrocytes, malaria parasites digest hemoglobin to provide growing space and to obtain nutrients. This process releases toxic by-products, inducing oxidative stress on the cell [2].

Approximately 160 genetic variants causing clinical deficiency of G6PD have been characterized [2]. The geographical distribution of these deficiency alleles closely reflects populations exposed historically to endemic malaria. The G6PD gene is present on the long arm of the X chromosome (Xq28) and consists of 13 exons with a length of 18 kilobases [3]. The active form of the G6PD enzyme is either a dimer or a tetramer of a single polypeptide subunit of about 59 kilodaltons [4]. G6PD deficiency is mainly found in populations originating from tropical and sub-tropical areas of the world, and geographic distribution is similar to that of falciparum malaria. This deficiency is beneficial as it is known that red cells that are deficient in G6PD are resistant to* Plasmodium falciparum (P. falciparum)* invasion since the parasite requires the enzyme for its normal survival in the host cell [1,5]. This deficiency offers selective protection against* P. falciparum* [6]. Trends of G6PD deficiency in *Plasmodium vivax (P. vivax)* endemic areas have been less revealing, with a weak association between the two being reported from around the world [7-9].

Three G6PD genotypes are common: G6PD B, G6PD A(+), and G6PD A(-). G6PD A(+) is characterized by a mutation at nucleotide 376, resulting in an Asparagine→Aspartic Acid (Asn→Asp) mutation that increases electrophoretic mobility of the enzyme [10,11]. G6PD A(-) shares the mutation at nucleotide 376 with G6PD A(+) and has a second mutation that produces enzyme instability and, therefore, deficiency. Four different such second mutations have been found, three of them producing the G6PD A(-) phenotype [12,13]. Although G6PD A(-) is thus genetically heterogeneous, the vast majority of subjects with G6PD A(-) contain a second mutation at nucleotide 202 (Valine→Methionine) and are therefore designated G6PD A(-)202A/376G [12].

*P. vivax *remains the second most common cause of clinical malaria (after *P. falciparum*). Globally, *P. vivax* malaria burden has decreased from 24·5 million cases in 2000 to 14·3 million cases in 2017 on account of improved global health efforts and international funding to fight malaria [14,15]. In 2014, 380,000 cases of *P. vivax* malaria were reported in India [14]. However, research directed at understanding the morbidity patterns and efforts to curb it remain underwhelming, especially compared to those for falciparum malaria [14,15]. North East India consists of eight states with an estimated population of 45,587,982 and contributes substantially to India’s vivax malaria burden [16]. In the region, malaria epidemiology is complicated by the high aboriginal population, varied terrain, rich forest cover, and climatic conditions favorable for the transmission of *Plasmodium* [17].

To the best of our knowledge, no previous literature has reported the frequency and involved genotypes of G6PD deficiency in patients with* P. vivax* malaria in this region. Considering the above, we undertook the present study to understand: (1) the frequency of G6PD deficiency in patients treated for* P. vivax* malaria in a tertiary care hospital in North East India, and (2) the most prevalent genotype of G6PD deficiency among the population seeking treatment for* P. vivax *malaria.

## Materials and methods

This hospital-based cross-sectional study was carried out between May 2014 and September 2016 after obtaining clearance from the institute's ethics committee (No. NEIGRIHMS/Micro/IEC-II/197/2012-13) dated 1st June 2012.

Eighty individuals diagnosed with* P. vivax* malaria through thin peripheral blood smear (PBS) using Leishman stain were included in the study. Individuals with a history of malaria or undiagnosed fever of more than 21 days duration were excluded. Patients with severe metabolic derangements and organ dysfunction evident in fasting blood glucose, serum creatinine, serum electrolytes, and liver function tests were also omitted from the study.

Sample collection and processing 

Written consent was obtained from all participant subjects after communicating to them the exact nature of the study. Under aseptic & antiseptic precautions, 3 ml of venous blood was collected in ethylenediaminetetraacetic acid (EDTA) vial from the median cubital vein. A thin peripheral blood smear was also obtained. The EDTA mixed blood was sent to the Department of Biochemistry and a peripheral blood smear to the Department of Microbiology for further necessary diagnostic processing. 

After collection, samples underwent enzyme assay for G6PD deficiency by the kinetic method using reagents supplied by Coral Clinical System (Tulip Diagnostics, Goa, India). G6PD in the red blood cells was released using a lysing agent (G-Six Kit, Coral Clinical System). The released G6PD catalyzed the oxidation of glucose 6-phosphate with the reduction of nicotinamide adenine dinucleotide phosphate (NADP) to NADPH. The rate of reduction of NADP to NADPH was measured as an increase in absorbance at 340 nm, proportional to the G6PD activity in the sample.

The EDTA samples with deficient phenotype were then centrifuged again to obtain the buffy coat and plasma. From the centrifuged sample, 50 µl buffy coat was used to isolate DNA. DNA was extracted with proteinase K digestion using a genomic DNA isolation kit (Qiagen India Pvt. Ltd., New Delhi, India). Approximately 1 μg of genomic DNA was amplified using primers 5′-GTCTTCTGGGTCAGGGAT-3′ (forward) and 5′-GGAGAAAGCTCTCTCTCC-3′ (reverse). Denaturation at 94°C for two minutes was followed by 45 cycles of denaturation at 94°C for 30 seconds, annealing at 60°C for 30 seconds, extension at 72°C for 60 seconds, and a final extension at 72°C for four minutes. The amplified products were digested with the restriction endonuclease Nla III & Fok I. The digested products were then subjected to horizontal agarose electrophoresis for the separation of digested fragments. Ethidium bromide-stained gels were then subjected to gel documentation apparatus (Labindia Analytical Instruments Pvt. Ltd., New Delhi, India) to study the fragments for polymorphism.

The Fok I and Nla III sites were detected using the appropriate endonucleases. All the G6PD enzyme deficient samples were examined for the nucleotide 376 A→G mutation, which produces a new Fok I cleavage site. Samples without this site were classified as G6PD B. Those containing the nucleotide 376 mutation were further examined for the G6PD deficient A(-) mutation, guanine→adenine (G→A), at nucleotide 202 by restriction analysis with Nla III. The samples containing the Nla III site were designated A(-), and those without it as A(+).

Data analysis

Data were recorded in Microsoft Excel (Microsoft® Corp., Redmond, WA) and were presented as counts or percentages. The prevalence of G6PD deficiency was calculated as the total number of subjects with deficient G6PD phenotype over the total of vivax malaria subjects in the study.

## Results

**Figure 1:**
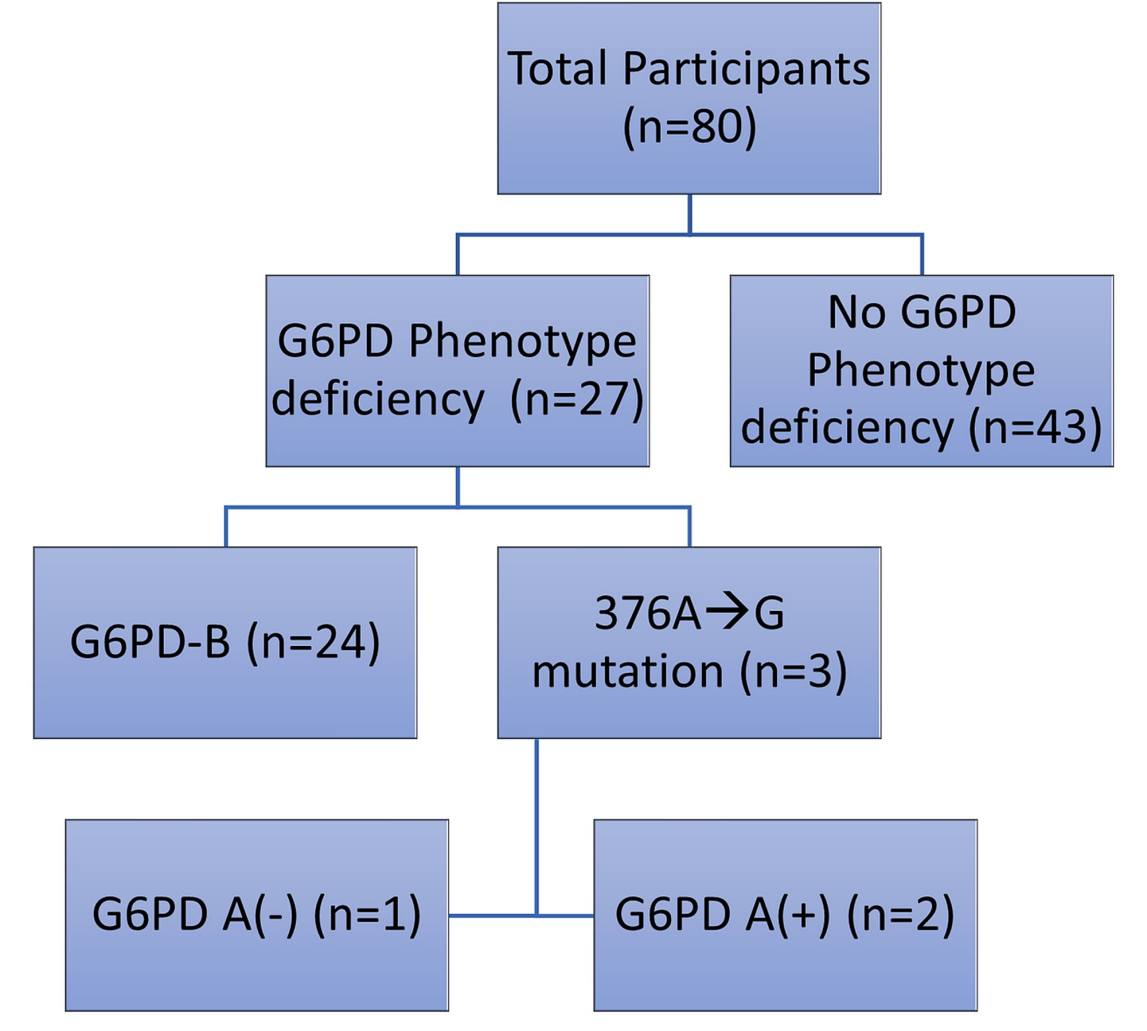
Frequency of G6PD deficiency and G6PD B and G6PD (A+/A-) genotypes G6PD: glucose-6-phosphate-dehydrogenase

Individuals with *P. vivax* malaria, as diagnosed by thin PBS using Leishman stain, were recruited for the study. Out of 80 individuals, 27 (33.75%) had a phenotypic deficiency of G6PD (Figure 1).

G6PD B genotype was the most common (n=24), accounting for 88.88% of all cases with G6PD deficiency.

**Figure 2:**
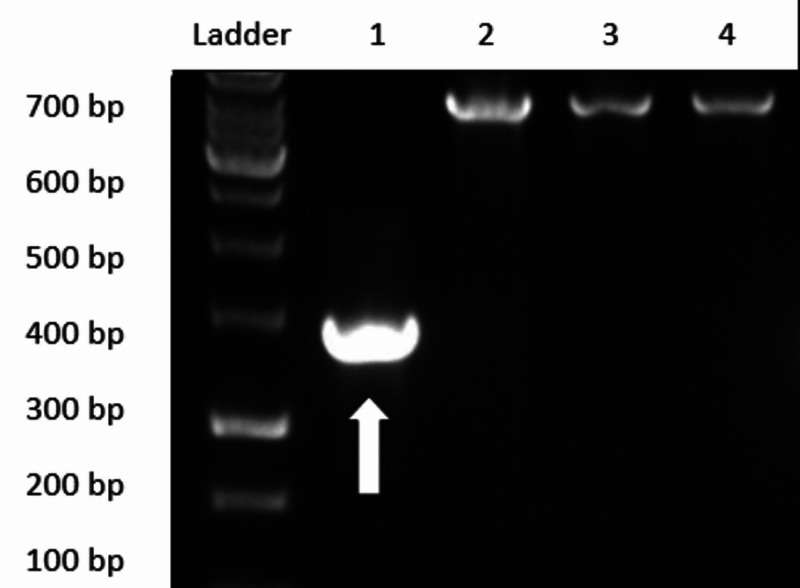
Agarose gel electrophoresis showing Fok I restriction site indicating 376 A→G mutation (white arrow) bp: base pair

**Figure 3:**
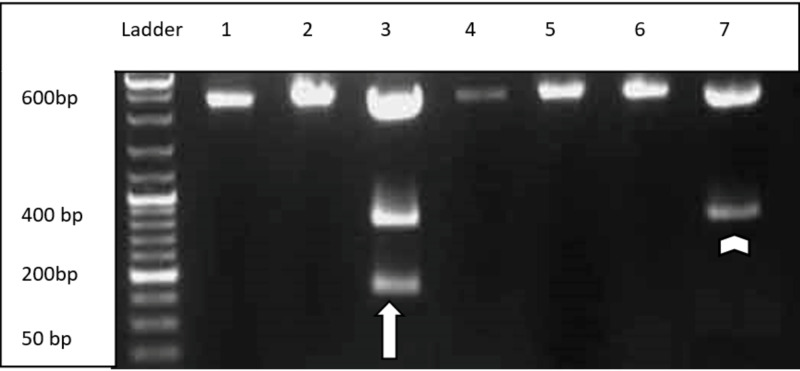
Agarose gel electrophoresis showing FoK I and Nla III restriction site indicating 376 A→G mutation (white arrowhead) and G6PD A(-) 202A/376G mutation (white arrow) bp: base pair

Polymerase chain reaction (PCR) followed by agarose gel electrophoresis, as shown in Figures 2, 3, confirmed three individuals to have Asn→Asp mutation at position 376 (A→G) phenotypically. Out of these three, one had an additional mutation at position 202 (G→A), indicating G6PD A(-)202A/376G genotype (Figure 3). 

## Discussion

North East India bears a large case burden of malaria in India and is co-endemic for both *P. falciparum* and *P. vivax* malaria [17-20]. Vivax malaria has been reported in almost all states of North East India, attributing up to 60-80% of all malaria cases in some states [17]. G6PD deficiency often follows geographic distribution similar to that of falciparum malaria, but studies associating G6PD deficiency and *P. vivax* malaria distribution are sparse [1,7-9,21].

One notable study by Kar et al. carried out among the Ao Naga tribe of North East India found that G6PD deficient female heterozygotes and male hemizygotes were protected against both *P. falciparum and P. vivax* malaria [22]. Saha et al. reported G6PD deficiency frequency of 3%-8% in different tribes of North East India [23]. About a third of the patients with *P. vivax* malaria in our cohort had G6PD deficiency. G6PD deficiency in India has been reported to be higher in the East India zone, the Himalayan region, and populations belonging to scheduled tribes [21]. Our study population checks all three of these categories and could explain the high percentage of G6PD deficiency. So far, seven G6PD variants have been described from India, but it is worth noting that these have been described from populations in other regions, and none have been characterized from North East India.

G6PD B is the most common allele in all populations of G6PD deficiency, accounting for 60-80% of all cases [4,21]. Unlike G6PD A(+) and G6PD A(-), G6PD B has normal enzymatic activity [4]. In our study, G6PD B was by far the most common type accounting for almost 90% of individuals with G6PD deficiency. G6PD B itself has 34 different reported mutations, which are widely distributed in different exons (Table 1) [24]. Sukumar et al. found G6PD Mediterranean (Serine→Phenylalanine) to be the most common variant among Indian caste groups [25]. This variant has 3% of normal enzymatic activity, and its frequency has been reported between 2% to 20% [4].

**Table 1:** Common G6PD genotypes with associated mutations A→G: adenine→guanine, G→A: guanine→adenine, G6PD: glucose-6-phosphate dehydrogenase

Genotype	Fok I restriction cleavage	Base mutation at 376	Amino acid substitution	Nla III restriction cleavage	Base Mutation at 202	Amino acid substitution
G6PD B	Negative	-	-	Negative	-	-
G6PD A+	Positive	A→G	Asparagine→Aspartate	Negative	-	-
G6 PD A-	Positive	A→G	Asparagine→Aspartate	Positive	G→A	Valine→Methionine

G6PD A(+) genotype corresponds to 90% enzymatic activity and is produced by mutation of adenine to guanine in position 376 resulting in the replacement of Asn by Asp [2]. Its frequency is estimated to be 15-40% [4]. Two patients in our study had G6PD A(+) mutation, corresponding to a frequency of 7.40%.

G6PD A(-) mutation results in 12% of normal G6PD activity and affects up to 25% of the population in Sub-Saharan Africa [26]. In our study, one patient (1.25%) had G6PD A(-) mutation. Although polymorphic variant of G6PD A(-) remains asymptomatic in a vast majority of individuals, episodes of acute hemolytic anemia may be precipitated by triggers causing oxidative stress (including primaquine used for the treatment of *P.vivax*) [26,27]. Devendra et al. found G6PD A(-) to be the predominant mutation (91.8%) in a study carried out in the southern part of India [28]. A better understanding of this geographic variance in genotypes within the same country is essential and warrants further research.

There were certain limitations to our study. The small size recruited from a single center is the chief limitation of our study and may affect generalizability. Genotypic mapping based on demographic and geographic characteristics of the sample population would have likely yielded novel and informative results but could not be carried out due to logistic difficulties.

In India, the national drug policy on malaria (2013) recommends treating vivax malaria with primaquine for 14 days but disallows its use in individuals with G6PD deficiency [29]. Although alternative dosing schedules have recently shown promise, data remains scarce, and national guidelines have not yet incorporated these regimens [29,30]. Since our study reports a high proportion of G6PD deficiency in patients of vivax malaria, epidemiologic and genotypic characterization may determine the rationale for G6PD deficiency screening of malaria patients receiving primaquine therapy. Furthermore, screening may also reveal insightful differences in G6PD deficiency genotypes for populations living in sub-tropical and alpine climates of the region.

## Conclusions

Malaria has historically been a major cause of mortality and morbidity in the north-eastern region of India due to factors conducive to the transmission of *Plasmodium.* About a third of patients in our study with *P. vivax *malaria had underlying G6PD deficiency, which was observed to be quite high. G6PD B was the most common variant, although a few cases with G6PD A(+) and G6PD A(-) variants were also noted. Screening of individuals for G6PD deficiency prior to initiation of primaquine therapy may avoid potential episodes of acute hemolysis. 

Further studies elucidating the geographic and genotypic characteristics of patients from the region may reveal or refute the association of G6PD deficiency and protection against severe *P. vivax *malaria and pave the way for consideration of guidelines for routine screening for G6PD deficiency in such patients.
